# The characteristics and pre-hospital management of blunt trauma patients with suspected spinal column injuries: a retrospective observational study

**DOI:** 10.1007/s00068-016-0688-z

**Published:** 2016-06-08

**Authors:** J. T. Oosterwold, D. C. Sagel, P. M. van Grunsven, M. Holla, J. de Man-van Ginkel, S. Berben

**Affiliations:** 1 School of Nursing and Health, University Medical Centre Groningen, PO Box 30.001, 9700 RB Groningen, The Netherlands; 2 Ambulance Department, University Medical Centre Groningen, Roden, The Netherlands; 3 Ambulance Emergency Medical Service Gelderland-Zuid, Nijmegen, The Netherlands; 4 Department of Orthopaedic Surgery, Radboud University Medical Centre, Nijmegen, The Netherlands; 5 Department of Rehabilitation, Nursing Science and Sports, University Medical Centre Utrecht, Utrecht, The Netherlands; 6 Faculty of Medicine, Clinical Health Sciences, Utrecht University, Utrecht, The Netherlands; 7 Eastern Regional Emergency Healthcare Network & IQ Scientific Centre for Quality of Healthcare, Radboud University Medical Centre, Nijmegen, The Netherlands; 8 Department of Critical and Emergency Care, Knowledge Centre of Sustainable Healthcare, HAN University of Applied Sciences, Nijmegen, The Netherlands

**Keywords:** Blunt trauma, Pre-hospital, Spinal column injury, Spinal immobilisation, Emergency medical services, Retrospective observational study

## Abstract

**Background:**

Pre-hospital spinal immobilisation by emergency medical services (EMS) staff is currently the standard of care in cases of suspected spinal column injuries. There is, however, a lack of data on the characteristics of patients who received spinal immobilisation during the pre-hospital phase and on the adverse effects of immobilisation. The objectives of this study were threefold. First, we determined the pre-hospital characteristics of blunt trauma patients with suspected spinal column injuries who were immobilised by EMS staff. Second, we assessed the choices made by EMS staff regarding spinal immobilisation techniques and reasons for immobilisation. Third, we researched the possible adverse effects of immobilisation.

**Design:**

A retrospective observational study in a cohort of blunt trauma patients.

**Study method:**

Data of blunt trauma patients with suspected spinal column injuries were collected from one EMS organisation between January 2008 and January 2013. Coded data and free text notes were analysed.

**Results:**

A total of 1082 patients were included in this study. Spinal immobilisation was applied in 96.3 % of the patients based on valid pre-hospital criteria. In 2.1 % of the patients immobilisation was not based on valid criteria. Data of 1.6 % patients were missing. Main reasons for spinal immobilisation were posterior midline spinal tenderness (37.2 % of patients) and painful distracting injuries (13.5 % of patients). Spinal cord injury (SCI) was suspected in 5.7 % of the patients with posterior midline spinal tenderness. A total of 15.8 % patients were immobilised using non-standard methods. The reason for departure from the standard method was explained for 3 % of these patients. Reported adverse effects included pain (*n* = 10, 0.9 %,); shortness of breath (*n* = 3, 0.3 %); combativeness or anxiety (*n* = 6, 0.6 %); and worsening of pain when supine (*n* = 1, 0.1 %).

**Conclusion/recommendation:**

Spinal immobilisation was applied in 96.3 % of all included patients based on pre-hospital criteria. We found that consensus among EMS staff on how to interpret the criterion ‘distracting injury’ was lacking. Furthermore, the adverse effects of spinal immobilisation were incompletely documented in pre-hospital care reports. To provide validated information on potential symptoms of SCI, a uniform EMS scoring system for motoric assessment should be developed.

## Introduction

Patients who have suffered blunt trauma resulting in spinal column injuries, such as spinal fractures or dislocations, are at risk of developing iatrogenic spinal cord injury (SCI) due to physical movement or manipulation [1–5].

SCI is defined as a traumatic injury to the spinal cord that results in loss of motor and/or sensory functions [6]. In a European cohort (*n* = 250,584) of severely injured trauma patients (excluding penetrating injuries), 13.2 % of immobilised patients had vertebral column injuries and 1.8 % sustained a SCI [7, 8]. It has been postulated that spinal immobilisation by emergency medical services (EMS) is required for all patients with suspected vertebral column injuries to prevent SCI after blunt trauma [1].

According to the 8th edition of the Advanced Trauma Life Support (ATLS) guidelines, spinal immobilisation should be maintained by a rigid cervical collar in combination with head blocks strapped to a spine board [9]. In the Netherlands, the EMS spinal immobilisation guidelines have been adjusted in 2002 and 2006 in accordance with the Pre Hospital Trauma Life Support (PHTLS) guidelines [10, 11]. According to the guidelines of 2006, full spinal immobilisation is only indicated in patients who have sustained blunt trauma and show one or more of the following symptoms: neck/back pain or tenderness, altered level of consciousness, neurological deficits and evidence of intoxication or painful distracting injuries. Departure from the guidelines is allowed, however, in case of neck muscle spasms, increased pain, increase of neurological deficits, signs of increased intracranial pressure (ICP) or combativeness/resistance of the patient [11]. In these situations, the EMS staff can opt for a rigid collar only or head blocks with spine board only.

Despite the assumed beneficial effect of the method of spinal immobilisation advocated by the ATLS guidelines, there is growing evidence that immobilisation is associated with severe adverse effects including serious respiratory problems [11–13], increased ICP [14–16], delirium [17], iatrogenic pain or discomfort [18, 19] and possible deterioration of SCI [2, 20–23]. Furthermore, spinal immobilisation can cause a delay in transportation time to the hospital, which can negatively influence outcomes in patients with SCI [24].

A limitation of these studies is that they were mainly hospital-based and lacked a full pre-hospital description of, for example, patient characteristics, immobilisation techniques and adverse effects of spinal immobilisation. There is a paucity of data on the characteristics of patients who received pre-hospital spinal immobilisation and on the adverse effects of immobilisation that may occur during this phase. Furthermore, it is unknown whether EMS staff follows the 2006 spinal immobilisation guidelines with regard to applied techniques when taking care of patients with spinal column injuries. Our study had three main research goals. First, we aimed to determine the pre-hospital characteristics of blunt trauma patients with suspected spinal injuries that were immobilised by EMS staff. Second, we assessed the reasons for spinal immobilisation and the choices made by EMS staff regarding spinal immobilisation techniques. Third, we researched the occurrence of possible adverse effects of immobilisation during the pre-hospital phase.

## Materials and methods

### Study design

A retrospective observational study was performed in a cohort of blunt trauma patients.

### Population and setting

The EMS of the region Gelderland-Zuid (VRGZ), the Netherlands, provides care to approximately 545,000 inhabitants from eight ambulance stations. All the 21 ambulances of VRGZ are staffed with two EMS professionals: an ambulance nurse and an ambulance driver. They will be further referred to as EMS staff in this study. They annually respond to approximately 21,000 high priority emergency ambulance calls. After every patient transport the EMS staff fills in a datasheet, which is added to the electronic patient record (EPR) of the EMS VRGZ.

### Data collection

All patients from the EPR of the EMS VRGZ who were transported between 1 January 2008 and 31 December 2012 and who met the inclusion criteria described in Table 1 were included in this study. There were no changes in the spinal immobilisation protocol during the study period. The study did not require an ethical approval because of the retrospective observational design from anonymised data. The data was provided by the medical manager of VRGZ to the authors in a Microsoft Excel spreadsheet.

**Table 1:** Inclusion criteria of patients (see “Material and methods”)

18 years or older
*and*
Life-threatening response call with a lights and sirens ambulance response
*and*
Blunt trauma
*and*
Full or partial external immobilisation
*and*
Ambulance transport from accident site to the Emergency Department

### Study population

Trauma patients with full or partial external immobilisation after blunt trauma who received a high priority of the ambulance dispatch centre were included in the study. High priority was defined as an ambulance being on site within 15 min after the initial contact with the emergency medical dispatcher using lights and siren.

Further inclusion criteria for the patients were that patients had to be ≥18 years of age and transported to either the Radboud University Hospital Nijmegen (Level 1 trauma centre) or the Canisius Wilhelmina Hospital Nijmegen (Level 2 trauma centre). Exclusion criteria were inter-hospital transport and helicopter emergency medical service (HEMS) transport.

### Variables and measurements

#### Characteristics of the included patients and nature of the incidents

Age, gender and alcohol use of the patients were included in the analysis. The nature of the incident was determined as traffic, home, sports, work-related and other.

#### Characteristics of time intervals

To determine the time patients were lying on the spine board before arrival at the Emergency Department (ED), the following time intervals were measured: on-scene time (OST), transportation time (TrT) and the total time (TT) from the initial dispatch until arrival at the hospital. Time values were written in the form hh:mm:ss.

#### Characteristics of suspected injury

Suspected injuries to the spinal column or spinal cord that were coded by EMS staff were determined. To define signs of high ICP, free text notes were screened for the following items according to the PHTLS literature: evidence of head trauma combined with pupillary changes (sluggish or non-reactive), hemiparesis, hemiplegia or a Glascow Coma Scale (GCS) <14 [10]. Evidence of possible cranial bleeding was derived from the data that was coded as ‘intracranial injury’ or ‘subarachnoid haemorrhage’.

#### Characteristics of consciousness and pain

Consciousness was measured with the GCS and categorised into three groups: severe (GCS 3–8), moderate (GCS 9–12) and mild (GCS 13–14) [10]. The general level of pain experienced by the patient was measured with scores on the Numeric Rating Scale (NRS) [25]. This is an 11-point scale ranging from 0 to 10. A score between 1 and 3 points was classified as mild pain, a score between 4 and 6 points as moderate pain and a score between 7 and 10 points as severe pain.

#### Characteristics of administered analgesics

The types of analgesics administered to the patients were recorded.

#### Characteristics of reasons for spinal immobilisation

**Fig. 1:**
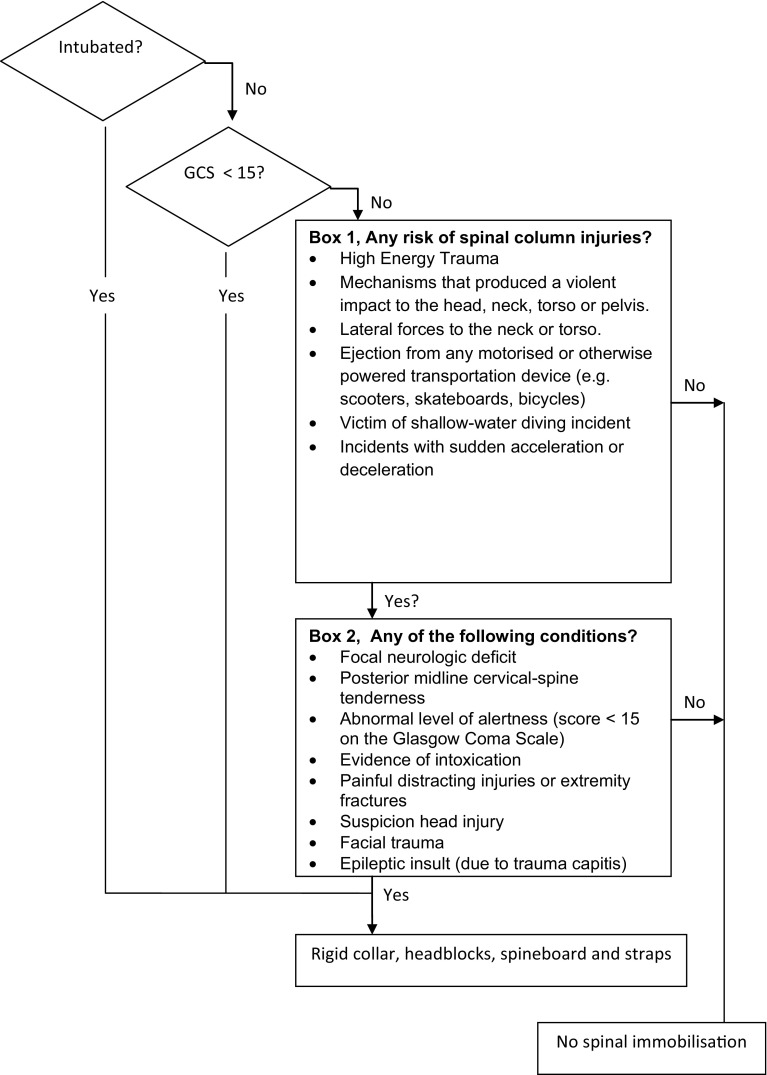
Dutch National Protocol Ambulance Care 2006 (derived from the Pre-hospital Spinal Immobilisation Guidelines 2002)

Spinal immobilisation is indicated when patients meet criteria as mentioned in box 1 (Fig. 1, box 1) combined with a criterion from box 2 (Fig. 1, box 2) [11]. The pre-hospital distinction between a contusion, luxation and/or fracture is not always reliable and valid; therefore, we chose to combine all the injuries that may cause acute functional impairment and subdivided them into the following categories: lower extremity pain, upper extremity pain, upper chest pain, abdominal pain, hip/iliac pain and a group with combined injuries from the previous categories. Patients with abrasions only were excluded.

The techniques used for spinal immobilisation were registered. The standard method of spinal immobilisation consists of the use of a rigid cervical collar, a long backboard, head blocks and straps. The following departures from the standard method were discerned: backboard and straps only, rigid collar only, vacuum mattress and rigid collar only, manual fixation of the head and neck only and scoop stretcher and rigid collar only. The reasons given by EMS staff for departure from the standard method were also noted [10].

Spinal immobilisation outside the standard guidelines refers to full spinal immobilisation of patients only based on the mechanism of injury (Fig. 1, box 1). These patients did not meet the criteria mentioned in box 2.

#### Adverse effects

All known adverse effects of spinal immobilisation that could be measured and detected by the EMS staff in the pre-hospital setting were registered. These effects include pain or discomfort as a result of spinal immobilisation [18], shortness of breath and a subsequent reduction in respiratory function [26] as a result of lying supine, vomiting or nausea in a moving ambulance and combativeness.

### Data analysis

Coded categorical data were presented in absolute numbers and percentages. The mean and standard deviations (SD) were calculated for continuous variables. Measured time was presented in minutes and seconds. Manifest content analysis was used to analyse the free text notes [27]. This quantitative research method was used to count the frequency of the following pre-defined characteristics reported in the free text: use of alcohol, symptoms of high intracranial pressure, nausea or emesis, adverse effects of spinal immobilisation, methods of spinal immobilisation and criteria for spinal immobilisation.

Differences between groups were calculated with Chi-square for categorical data, and one-way ANOVA was used to test differences between several groups. P values of ≤0.05 were considered as significant for all tests. IBM SPSS statistics version 20.0 was used to analyse the data.

## Results

A total of 1082 patients received spinal immobilisation in the region Gelderland-Zuid between January 2008 and December 2012. A total of 654 (60.4 %) patients were transferred to a Level 1 trauma centre and 428 (39.6 %) patients to a Level 2 trauma centre.

### Characteristics of the included patients and type of incident

The mean age of the included patients was 43 years [SD ± 18.3 (range 18–93 years)]. Patients aged 65 years and older represented 14 % (*n* = 151) of the total study population. The male–female ratio was 643–439 (59–41 %). Alcohol use was described by EMS staff in 129 (11.9 %) patients. Crash/collision incidents were coded in 209 (19.3 %) patients as the cause of injury. In 754 (69.7 %) patients the type incident was not coded by EMS staff.

### Characteristics of time interval

The mean on scene time (OST) was 25.33 (SD 10.22) and mean transport time (TrT) was 14.24 (SD 8.14) (Table 4). The mean total time (TT) between initial contact with the emergency dispatcher and the arrival at the hospital was 49.13 (SD 16.25).

### Characteristics of suspected injury

In 402 (37.2 %) patients the EMS staff suspected a spinal column injury, and 62 (5.7 %) patients demonstrated symptoms of SCI. Other suspected injuries are described in Table 2. Signs of increased ICP were documented in 75 (6.9 %) patients.

**Table 2:** Patient demographics and characteristics

	*n*	%	Pain assessment performed by EMS staff at arrival			
			*n*	%	Mean NRS^a^	95 % CI for mean
All patients	1082	100	311	28.7	2.09	1.73–2.45
Gender						
Male	643	59.4	188	17.4	2.03	1.58–2.48
Female	439	40.6	123	11.4	2.18	1.6–2.76
Age						
18–64 (mean, SD)	931 (43, 18)	86.0	279	25.8	2.11	1.73–2.49
≥65	151 (74, 7)	14.0	32	3.0	1.88	0.75–3.01
GCS at arrival EMS						
GCS 3–8	61	5.6	–	–	–	–
GCS 9–13	66	6.1	14	1.3	2.14	0–4.28
GCS 14–15	919	84.9	271	25.0	2.07	1.7–2.44
Type of accident						
Traffic	209	19.2	56	5.2	2.18	1.25–3.11
Home	66	6.0	17	1.6	2.65	0.86–4.44
Sports	17	1.6	8	0.7	2.75	0.94–4.56
Work	28	2.6	7	0.6	0.86	0.31–2.03
Other	8	0.7	4	0.4	0.50	0.48–1.48
Alcohol use	129	11.9	–	–	–	–
18–30 years	37	3.4	–	–	–	–
31–64 years	81	7.5	–	–	–	–
≥65 years	11	1.0	–	–	–	–
Nausea or vomiting	87	8.0	–	–	–	–
Injuries^b, c^						
Suspected vertebral column injury	402	37.2	–	–	–	–
Suspected spinal cord injury	62	5.7	–	–	–	–
Head injury	407	37.6	–	–	–	–
Signs of increased ICP	75	6.9	–	–	–	–
No signs of increased ICP	326	30.1	–	–	–	–
Jaw fracture	6	0.6	–	–	–	–
Upper torso injury	174	16.1	23	2.1	2.4	–
Abdominal injury	40	3.7	3	0.3	8	–
Lower extremity injury	74	6.8	11	1.0	3.36	–
Upper extremity injury	31	2.8	3	0.3	6	–
Injury to different body parts	–	–	12	1.1	2.7	–

^a^Numeric Rating Scale

^b^There may be more than one injury in a patient

^c^Any other injury with the exception of abrasions

### Characteristics of consciousness and pain

Severe loss of consciousness (GCS 3–8) at arrival of the ambulance was found in 61 (5.6 %) patients and moderate loss of consciousness (GCS 9–12) in 66 (6.1 %) patients. A total of 919 (84.9 %) patients were alert (GCS of 14 or 15). Scores of 36 (3.3 %) patients were missing.

In 771 (71.3 %) patients the EMS staff did not report the NRS. The pain intensity reported by the remaining 311 (28.7 %) patients at arrival of the ambulance varied: 200 (18.5 %) patients reported no pain, 24 (2.2 %) mild pain, 37 (3.4 %) moderate pain and 50 (4.6 %) severe pain. Mean pain score (NRS) in the study population was 2.09. A second assessment of pain intensity (at arrival at ED) was missing in 1008 (93.2 %) patients and therefore excluded from further analysis. Results of the pain assessment at arrival of the EMS staff are presented in Table 2.

### Characteristics of administered analgesics

Analgesics of one type (fentanyl, ketamine, nitrous oxide/oxygen mixture or paracetamol) were given to 229 (21.2 %) patients; fentanyl and ketamine to 15 (1.4 %) patients; fentanyl and paracetamol to 19 (1.8 %) patients and a nitrous oxide/oxygen mixture with fentanyl to 2 (0.2 %) patients.

### Reasons for spinal immobilisation

The reasons for spinal immobilisation are described in Table 3. Midline tenderness of the spine after blunt trauma was the main reason for immobilisation (37.2 %, *n* = 402). Painful distracting injuries came second (13.5 %, *n* = 146). In case of a non-tender spine, upper torso injuries were considered most frequently as distracting injuries leading to full spinal immobilisation (5 %, *n* = 55). The mean NRS score of the group of patients that were immobilised based on the criterion ‘painful distracting injuries’ was 3.2 (*n* = 52) with a mode of 0 (Table 4). An analysis of variance showed that the mean pain scores on the different categories of injuries revealed a statistically significant main effect, Welch’s *F*(5, 6.731) = 7.5, *P* = 0.0011, indicating that not all of the injuries had the same average pain score. Post hoc comparisons, using Games-Howell post hoc procedure, showed that patients with abdominal injuries (*M* = 8.00, SD = 1.00) had a significantly higher average pain score than patients with upper torso injuries (*M* = 2.43, SD = 3.15) and patients with injuries to different body parts (*M* = 2.67, SD = 3.96).

**Table 3:** Characteristics of pre-hospital emergency care

	*n*	%	Mean (SD)
Time intervals			
Time on scene^a^	1055		25′33″ (10′22″)
Time transport to ER^b^	1034		14′24″ (8′14″)
Time from dispatch to ER	1080		49′13″ (16′25″)
Administered analgesics			
Fentanyl	199	18.4	
Esketamine	21	1.9	
Nitrous oxide/oxygen mixture	0	0	
Paracetamol	9	0.8	
Fentanyl and ketamine	15	1.4	
Fentanyl and paracetamol	19	1.8	
Fentanyl, paracetamol and ketamine	2	0.2	
Fentanyl and nitrous oxide/oxygen	2	0.2	
Spinal immobilisation according to the applicable guideline	1059	97.9	
Posterior midline spine tenderness, and/or abnormal level of alertness (GCS < 15), and/or focal neurological deficit, and/or facial trauma, and/or epileptic insult (due to trauma capitis)	767	70.9	
Painful distracting injuries only	146	13.5	
Evidence of intoxication	129	11.9	
Unknown	17	1.6	
Spinal immobilisation outside the applicable guidelines	23	2.1	
No injury, only based on trauma mechanism	23	2.1	
Method of spinal immobilisation			
Full spinal immobilisation	911	84.2	
Rigid collar only	55	5.1	
Spine board with straps only	102	9.4	
Semi rigid brace (KED^®^) only	2	0.2	
Semi rigid brace (KED^®^) with rigid collar	4	0.4	
Vacuum mattress with rigid collar	6	0.6	
Scoop stretcher only	1	0.6	
Scoop stretcher with rigid collar	1	0.1	

^a^Cases falling greatly outside of the range (<5 or >60 min) were analysed by free text and, 27 cases were deleted because of incorrectness

^b^Cases falling greatly outside of the range (<60 s or >45 min) were analysed by free text, and 48 cases were deleted because of incorrectness

**Table 4:** Categories of painful distracting injuries in cases of non-tender spine and GCS 15

	*n* (%)	Number of NRS^a^ score	Mode NRS score	Mean NRS score	Min–max	
Categories of distracting injuries	146 (13.5)	52	0	3.21	0–10	*P* = 0.01^b^
Upper torso injury	55 (37.7)	23	0	2.4	0–8	
Injury to different body parts	38 (26)	12	0	2.6	0–10	
Lower extremity injury	26 (17.8)	9	0	3.7	0–10	
Hip, iliac injury	10 (6.8)	2	0, 3	1.5	0–3	
Upper extremity injury	9 (6.2)	3	0, 8, 10	6	0–10	
Abdominal injury	7 (4.8)	3	7, 8, 9	8	7–9	
Other	1 (0.7)	–	–	–	–	

^a^Numeric rating scale

^b^ *P* value from Welch test

Spinal immobilisation outside the standard guidelines was performed on 23 (2.1 %) patients. In these cases, the decision of the EMS staff to stabilise the spine was based only on the criteria shown in Fig. 1, box 1 (trauma mechanism). The remaining 236 (21.8 %) patients were immobilised based on one of the other criteria of the Dutch NPA: focal neurological deficits, evidence of intoxication, facial trauma, epileptic insult due to trauma capitis, or unknown.

In our study population, 911 (84.2 %) patients received full spinal immobilisation (rigid collar, head blocks, spine board and straps). A total of 102 (9.4 %) patients were immobilised by spine board and straps only. Two (0.2 %) patients were immobilised by a semi-rigid brace only that secures the head, neck and torso (Kendrick Extrication Device^®^). Four (0.4 %) other patients were immobilised by the semi-rigid brace in conjunction with a rigid cervical collar. The vacuum mattress in combination with the cervical rigid collar was used in 6 (0.6 %) patients. Transportation by means of scoop stretcher occurred once with the rigid collar (0.1 %) and once (0.1 %) without manual in-line stabilisation. The remaining 55 (5.1 %) patients were immobilised by a rigid cervical collar only (Table 3).

Departure from the standard method of spinal immobilisation was explained by EMS staff in 32 cases (3 %) (Table 5). Pain from surrounding injuries was the main reason for omitting the rigid cervical collar (*n* = 10, 0.9 %), followed by combativeness or anxiety (*n* = 7, 0.6 %). The choice for omitting the spine board was mainly based on shortness of breath (*n* = 2, 0.2 %). Following PHTLS guidelines, the rigid collar should be removed in case of increased ICP. In this study population the percentage of patients who were immobilised by backboard only (*n* = 102, 9.4 %) did not differ in patients with or without signs of increased ICP [*χ* ^2^ (1, *N* = 1082) = 1.141, *P* = 0.286].

**Table 5:** Characteristics of pre-hospital emergency care

Reasons for departure from the standard method of spinal immobilisation	*n*	%
Omitting the rigid collar	26	2.4
Pain from surrounding injury		
Sternum injury	1	
Clavicle fracture	5	
Ear injury	1	
Shoulder injury	1	
Jaw fracture	2	
Combativeness or anxiety	7	
Shortness of breath	2	
Non- fitting (anatomic or clothing)	6	
Lumbar pain/tenderness only	1	
Omitting the spine board	6	0.6
Shortness of breath	2	
Worsening of pain when supine	1	
Combativeness or anxiety	1	
Unclear	2	

### Adverse effects

Vomiting or nausea was described in 87 (8.0 %) patients. More than half of these patients (50.6 %) received the antiemetic metoclopramide. In 45 (4.5 %) patients antiemetic drugs were administered prophylactically. Other adverse effects of spinal immobilisation included pain (*n* = 10, 0.9 %,), shortness of breath (*n* = 3, 0.3 %) and anxiety/combativeness (*n* = 6, 0.6 %) (Table 6).

**Table 6:** Adverse effects of spinal immobilisation reported as free text by the EMS

	*n* (%)
Pain	10 (0.9)
Shortness of breath	4 (0.4)
Anxiety/combativeness	6 (0.6)
Worsening of pain when supine	1 (0.1)

There were no reports of progressive signs of SCI.

## Discussion

To our knowledge, this is the first study that gives a comprehensive overview of the characteristics and pre-hospital management of blunt trauma patients with suspected spinal column injuries.

Our first objective was to determine the pre-hospital characteristics of this category of patients. The most important findings were that the EMS staff suspected spinal column injuries in 402 (37.2 %) patients and that 62 (5.7 %) patients had symptoms of SCI. The EMS staff did not report the NRS in 771 (71.3 %) patients. Painful distracting injuries were found in 146 (13.5 %) patients. The standard method of immobilisation was not used in 171 (15.8 %) patients. Finally, in 22 (2 %) cases adverse effects were reported by the EMS. These included pain due to spinal immobilisation (*n* = 10, 0.9 %,); shortness of breath (*n* = 3, 0.3 %); combativeness or anxiety (*n* = 6, 0.6 %); and worsening of pain when supine (*n* = 1, 0.1 %).

Our second objective was to assess the reasons for spinal immobilisation and the choices made by EMS staff regarding spinal immobilisation techniques The reason for spinal immobilisation was clear to the EMS staff in most cases (*n* = 1059, 97.9 %). There was a significant mechanism of injury (Fig. 1, box 1) combined with specific signs and symptoms (Fig. 1, box 2). We found that consensus on the implementation of the criterion ‘distracting injury’ was lacking among EMS staff. A total of 146 (13.5 %) of the patients who did not have spinal tenderness after blunt trauma were immobilised because of a painful injury. There is, however, a difference between a painful injury and a distracting injury. While a distracting injury is a criterion for spinal immobilisation according to Fig. 1, box 2, this criterion was interpreted differently by the various EMS staff. A distracting injury is defined by PHTLS as follows: long bone fractures, visceral injury requiring surgical consultation, large laceration, degloving or crush injury, large burns and any other injury producing acute functional impairment (Table 7) [10]. Although the most painful injuries may be considered as ‘distracting’, we could not demonstrate an association between high NRS scores at arrival of the EMS staff and distracting injuries as a reason for spinal immobilisation. The mean NRS score in the group of patients that were immobilised based on the criterion ‘painful distracting injuries’ was 3.2 (*n* = 52) with a mode of 0.

**Table 7:** Distracting injuries according to the PHTLS guidelines, fifth edition, 2003

Long bone fractures
Visceral injury requiring surgical consultation
Large laceration
Degloving or crush injury
Large burns
Any other injury producing acute functional impairment

Previous studies by Hefferman et al. [28] and Domeier et al. [29] researched whether the definition of a distracting injury could be narrowed. Hefferman et al. focused on the c-spine and showed that patients with an upper torso injury, in cases of a non-tender cervical spine, might have sustained a cervical spine injury. Domeier et al. redefined the term ‘distracting injury’ as: ‘a suspected extremity fracture proximal to the wrist or ankle’.

There is increasing evidence that a distracting injury, as currently defined, does not affect the sensitivity of the physical examination. Konstantinides et al. [30] concluded that only the upper chest injuries may be significant enough to decrease the sensitivity of the physical examination of the cervical spine in alert and non-intoxicated patients blunt trauma patients. Furthermore, Dahlquist et al. [31] showed, that femur fractures should not be considered as distracting injuries for cervical spine assessment. Clinical examination is a sensitive screening method for thoracolumbar spine clearance in patients with distracting injuries [32].

Therefore, further research and clarification of the criterion ‘distracting injury’ or a narrowing of the definition (following Hefferman et al., Domeier et al.) based on pain scores combined with injury assessment is warranted. We believe this will ultimately lead to a reduction of adverse effects of spinal immobilisation and potentially to a decrease of the number of patients, that is unnecessarily exposed to X-rays.

In 23 (2.1 %) patients spinal immobilisation was based on the mechanism of injury criteria (Fig. 1, box 1). Departure from the standard method of spinal immobilisation was found in 171 (15.8 %) cases. A high CPI was not a reason for removal of the rigid collar, despite the fact that 75 (6.9 %) of our cohort had signs of increased ICP. No statistically significant differences could be found for the application of a rigid collar between groups of patients with or without signs of high ICP. The recommendation to remove the rigid collar in case of increased ICP was made by the PHTLS in 2002 and accepted the NPA in 2006. The reason that EMS staff did not depart from the standard method of spinal immobilisation in cases of high ICP may be explained by the non-explicit naming of the removal of the rigid collar in the NPA. A lack of awareness of this guideline can cause further increase in ICP [14]. The arguments used by EMS staff to depart from the standard method of spinal immobilisation were in accordance with the Dutch NPA protocol. One exception concerned a patient who was immobilised by spine board only because of complaints of lower back pain. The patient did not complain of pain in the cervical region. We found that the documentation of reasons for departure from the standard method was inadequate. It remains unclear, for instance, why 139 (12.8 %) patients were not immobilised based on the standard method. Departure from the standard method is permissible if the EMS staff can substantiate this departure. Patients and other healthcare providers have reason to expect that pre-hospital treatment follows current protocols or guidelines. It is therefore vital that the reasons for departure from the standard method are adequately documented by EMS staff. Adequate documentation leads to improved transparency of pre-hospital care and is necessary when the EMS staff or organisation has to account for its decisions [33].

The third objective of our study was to research the occurrence of possible adverse effects during the pre-hospital phase. Previous research has found adverse effects of spinal immobilisation during this phase. In their study on the effects of spinal immobilisation on healthy volunteers, Kwan et al. [18] found that 55 % of healthy volunteers complained of moderate to severe pain within 30 min after spinal immobilisation. In addition, Bauer and Kowalski [26] found that certain devices used for spinal immobilisation had restrictive effects on the pulmonary function in the healthy, non-smoking man. Since the average on-scene and transportation times exceeded 30 min, we expected to find adverse effects of spinal immobilisation. Our findings did not correspond with previous research, however, adverse effects were found in only 22 (2 %) cases (pain due to spinal immobilisation (*n* = 10, 0.9 %,); shortness of breath (*n* = 3, 0.3 %); combativeness or anxiety (*n* = 6, 0.6 %); and worsening of pain when supine (*n* = 1, 0.1 %). A possible explanation for this discrepancy could be that the pre-hospital time is too short for the occurrence of the described adverse effects. Another explanation could be that pre-hospital data were incompletely documented, which was frequently observed in our study. The chosen design (retrospective EPR study) may also underestimate the true rate of adverse effects [34]. Recently, there is a lot of debate and research ongoing in the call for alternative spinal motion restriction. There are many side effects known of spinal immobilisation, and researchers recently came up with evidence that cervical spine movement was up to four times as high during extrication by EMS as by controlled self-extrication [35]. To reduce the adverse effects caused by spinal immobilisation, the National Association of EMS physicians (NAEMSP) and the American College of Surgeons Committee on Trauma (ACS-COT) have published a position paper in 2013. In this paper they describe that the utilization of backboards for spinal immobilization during transport should be judicious and not be used at all times. A professional consideration should take into account the benefits as well as the risks [36].

Finally, we found a high number of patients with symptoms (determined by EMS staff) of SCI in our study compared with a large European cohort study (5.7 versus 1.8 %, respectively) [37]. Nevertheless, it is difficult to draw comparisons, for while EMS staff can detect symptoms of SCI such as muscle weakness, paralysis or altered sensation, numbness, tingling or loss of sensation in hands, fingers, feet or toes [38], it lacks a validated instrument to uniformly detect symptoms of SCI. The Emergency Department (ED) uses standards of the American Spinal Injury Association (ASIA-ICLOS) to assess damage to the spinal cord of patients. These standards are not applicable in the pre-hospital setting for practical reasons. Furthermore, the EPR of the EMS does not state that documenting symptoms of SCI is mandatory. This means the number of symptoms of SCI in the pre-hospital setting might be over- or underestimated in our study. For future research it is necessary to develop a validated and uniform instrument to measure symptoms of SCI in the pre-hospital setting. This enables clinicians to monitor symptoms of SCI over time and to see whether the patient shows signs of deterioration or improvement. This knowledge can contribute to the evaluation of the effectiveness of the current (pre) hospital spinal immobilisation guidelines and improve the quality of care.

This study has some limitations. Data were obtained from only one of the 25 EMS organisations in the Netherlands and may not be representative for spinal immobilisation care in other regions than Gelderland-Zuid. To overcome this limitation, we used a large sample size and we included both rural and urban areas. Despite the regional differences in training and education, all 25 EMS organisations follow a national protocol and their staff is certified by an independent national organisation (Dutch Ambulance Institute).

Another limitation is that we were not able to demonstrate the appropriate use of and adherence to the spinal immobilisation protocol as outlined in Fig. 1 because we did not include patients after blunt trauma who did not receive spinal immobilisation. Furthermore the final outcome of spinal immobilised patients is unknown. Only suspected cases are described and we were not able to compare the patients with suspected spinal column injury with patients in which actual vertebral column/cord injury was diagnosed.

A final limitation concerns the fact that data on a number of variables were incorrectly recorded or missing in the EPR. Adequate documentation by ambulance staff is not only vital for the provision of good care, but also important for trauma research [33, 39, 40]. The lack of adequate EMS documentation has been previously reported [40]. We tried to minimise information bias by analysing the coded data in combination with free text fields. Based on the results of our study, we recommend a revision of the EMS documentation protocol in order to improve the collection of data for research purposes.

## Conclusions

This retrospective observational study described the characteristics and pre-hospital management of patients who received spinal immobilisation by EMS staff. Suspicion of spinal column injuries was documented in 37.2 % of the patients and in 5.7 % of the patients EMS staff reported symptoms of SCI. The combination of a rigid collar, spine board with straps and head blocks (full spinal immobilisation) was used in 84 % of the patients. The remaining 16 % received an alternative or incomplete form of spinal immobilisation. Although one-third of the patients showed signs of head injury and 7 % of these patients had signs of increased ICP, we could not demonstrate the removal of the rigid collar in cases of increased ICP (as advised by PHTLS).

Evident complications of spinal immobilisation were described in less than 2 % of the patients by EMS staff. A reduction in the number of patients who require spinal immobilisation can be achieved by clarifying the term ‘distracting injury’. Pain scores were under-recorded by the EMS staff in this cohort.

Finally, attention should be given to improving the process of data registration to make data more reliable. Good documentation is fundamental to scientific research. Therefore, management and staff of the ambulance services should be encouraged to improve their digital records to contribute to future pre-hospital research.
